# Exploring Copaiba and Andiroba Oils: A Comprehensive Review of Composition, Physicochemical Properties and Pharmacological Activities in Advanced Delivery Systems

**DOI:** 10.3390/pharmaceutics18060642

**Published:** 2026-05-23

**Authors:** Ana Luisa Pinto Magalhães, Nayara Santana Peixoto Moura, Janaína de Alcântara Lemos, Carolina de Aguiar Ferreira, Danyelle M. Townsend, Juliana de Oliveira Silva, Anna Eliza Maciel de Faria Mota Oliveira, André Luis Branco de Barros

**Affiliations:** 1 Department of Pharmaceutical Technology, Faculty of Pharmacy, Federal University of Minas Gerais (UFMG), Belo Horizonte 31270-901, Minas Gerais, Brazil; analuisa.p.magalhaes@gmail.com (A.L.P.M.); nayaraspeixoto@gmail.com (N.S.P.M.); jana_alemos@hotmail.com (J.d.A.L.); julianaoliveira.far@gmail.com (J.d.O.S.); 2 Department of Pharmacology and Toxicology, Biomedical Engineering and Radiology, Michigan State University (MSU), East Lansing, MI 48824, USA; deaguia1@msu.edu; 3 Department of Drug Discovery and Biomedical Sciences, Medical University of South Carolina (MUSC), Charleston, SC 29425, USA; danyelle.m.townsend@gmail.com; 4 Department of Biological and Health Sciences, Federal University of Amapá (UNIFAP), Macapá 68903-419, Amapá, Brazil; annaeliza.maciel@gmail.com; 5 Department of Clinical and Toxicological Analyses, Faculty of Pharmacy, Federal University of Minas Gerais (UFMG), Belo Horizonte 31270-901, Minas Gerais, Brazil

**Keywords:** *Copaifera* spp., *Carapa guianensis*, nanosystems, nanoemulsion, nanocarrier, oleoresin, bioavailability

## Abstract

**Background/Objectives**: The convergence of traditional medicinal practices in Brazil’s vast biodiversity has fueled pharmaceutical interest in advancing plant-derived formulation. Copaiba (*Copaifera* spp.) and andiroba (*Carapa guianensis*) are central to both the economic landscape and healing traditions of the Amazon rainforest. Derivatives from these species have diverse applications, with their oils representing important raw materials for therapeutic use. However, the poor aqueous solubility of oils remains a major barrier to developing formulations with optimal bioavailability. Nanotechnology offers a strategic approach to address this limitation, as nanosystems improve stability, solubility, and biological performance. **Methods**: This narrative review compiles and analyzes contemporary literature on the chemical composition, physicochemical properties, and pharmacological activities of copaiba and andiroba oils, with emphasis on studies involving nanoformulations, aiming to overcome the solubility limitations of these oils. **Results**: Evidence from the literature indicates that nanoencapsulation enhances the anti-inflammatory, antimicrobial, and wound-healing activity of the oils’ main constituents, such as beta-caryophyllene and limonoids. However, inconsistencies in reported chemical composition and physicochemical properties across studies highlight the lack of standardized characterization and extraction methods, potentially hindering the development of reproducible nanosystems. **Conclusions**: Nanoencapsulation represents a promising strategy to improve the therapeutic potential of Amazonian oils. Nevertheless, further efforts are required to standardize methodologies and expand clinical studies to confirm the efficacy and safety of nanosystems derived from these natural products.

## 1. Introduction

The Amazon rainforest stands as one of the planet’s largest reservoirs of biodiversity, harboring a vast array of microorganisms, fauna, and notably flora, much of which remains scientifically unexplored [1,2]. For centuries, native communities have relied on medicinal plants for healthcare, passing down invaluable traditional knowledge that has attracted increasing attention from the pharmaceutical and biotechnology sectors [3,4,5]. Among the native species, copaiba (*Copaifera* spp.) and andiroba (*Carapa guianensis*) have drawn significant scientific and pharmaceutical interest due to their prominent roles in local medicine and industry. The oils derived from these species have a rich history of ethnopharmacological use among indigenous populations, reflecting millennia of accumulated empirical knowledge.

Copaiba oleoresin, rich in sesquiterpenes (mainly β-caryophyllene and α-humulene) and diterpenes, exhibits several biological activities, including anti-inflammatory, antimicrobial, antioxidant, tissue-regenerating, immunomodulatory, and antiparasitic effects [6,7,8,9]. These pharmacological properties have contributed to the growing interest in copaiba oil for pharmaceutical, veterinary, and nanotechnology-based applications. These recent findings reinforce copaiba oil’s versatility as a multifunctional bioactive agent and its value as a sustainable Amazonian product. In parallel, andiroba oil, extracted from the seeds, is distinguished by high content of bioactive fatty acids and limonoids, which confer well-documented anti-inflammatory [10,11,12], insecticidal [13,14,15], and potent wound-healing properties [16,17,18,19]. These activities are increasingly substantiated by both traditional medicinal use and cutting-edge biotechnological research.

Despite their considerable therapeutic potential, both oils are constrained by physicochemical challenges, particularly low aqueous solubility and the resulting low bioavailability, which limit their application in conventional pharmaceutical formulations. To address these hurdles, nanotechnology-based approaches, including nanoemulsions, nanostructured lipid carriers, and polymeric hydrogels, have emerged as effective approaches, enabling controlled release, targeted delivery, and improved cellular uptake. Building on this foundation, recent studies on research into nanoemulsion-based formulations highlight the therapeutic potential of copaiba and andiroba oil in health applications, with advanced delivery systems demonstrating enhanced efficacy and improved safety compared to crude preparations [20,21,22,23,24,25,26].

In this context, the present integrative review aims to consolidate current evidence on the chemical composition, physicochemical properties, and pharmacological activity of copaiba and andiroba oils, with a particular emphasis on advances and innovations provided by the application of nanosystems. The objective is to clarify the therapeutic potential of these Amazonian resources for modern medicine, advocate for the standardization of extraction and quality control methodologies, and identify key directions for research, clinical translation, and sustainable development.

## 2. Materials and Methods

### Review Methodology

This study was conducted as a narrative literature review, aiming to provide a comprehensive and critical overview of the scientific knowledge regarding the composition, properties, and nanoformulations of copaiba and andiroba oils.

The literature search was performed using the electronic databases PubMed, Web of Science, Scopus, and Google Scholar. The selection of studies was based on their relevance to the topics of interest, including chemical composition, physicochemical properties, biological activities, and the development of nanosystems involving copaiba and andiroba oils.

Relevant articles were selected based on their scientific contribution, methodological consistency, and alignment with the scope of this review. Additional references were identified through the analysis of reference lists of selected studies, when appropriate.

## 3. Results and Discussion

### 3.1. Composition of Andiroba and Copaiba Oils

**Figure 1:**
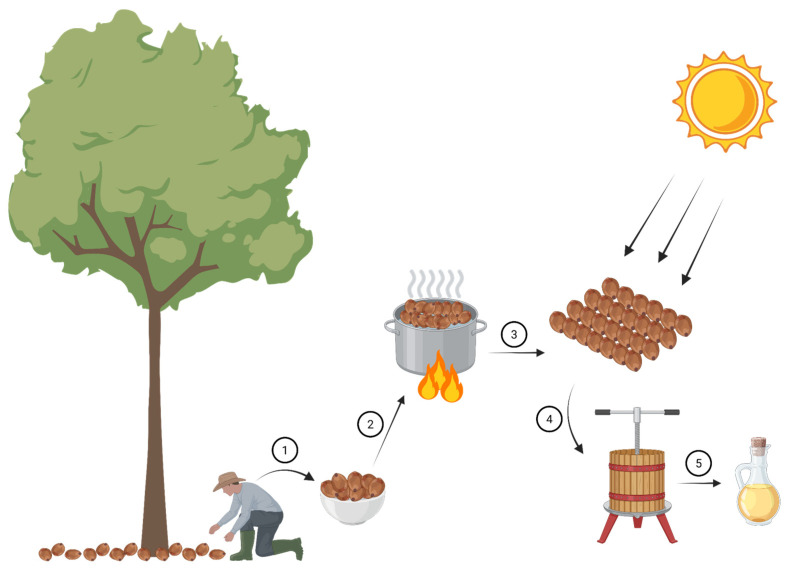
Traditional method for andiroba oil extraction. Step 1. Mature andiroba seeds are collected and selected for processing. Step 2. The seeds are cooked in heated water to facilitate the subsequent oil release. Step 3. After cooking, the seeds are dried under controlled conditions to reduce moisture content. Step 4. The dried seed mass is subjected to extraction procedures for oil recovery. Step 5. The obtained andiroba oil is collected and stored. (Created with BioRender.com.)

*Andiroba Oil.* Andiroba oil is extracted from the seeds of *Carapa guianensis* (*Meliaceae* family), a species widely distributed throughout the Amazon basin. The seeds are collected from mature fruits, typically by local communities utilizing traditional knowledge and low-impact harvest techniques. After collection, the seeds are cooked to soften them and facilitate the release of oil from the pulp. Subsequently, the seeds are sun-dried and then mechanically or cold-pressed to obtain the viscous, yellowish oil. This process preserves the integrity of its bioactive constituents while supporting regional economies through sustainable extractivist practices [27] (Figure 1).

Chemically, andiroba oil is composed of saponifiable substances (accounting for about 95%) that are mainly triacylglycerols containing fatty acids with high levels of unsaturation, such as linoleic acid, a polyunsaturated compound noteworthy in the oil’s profile. Saturated and monounsaturated fatty acids are also present, especially palmitic, stearic, and oleic acids [17,28]. However, the precise composition of *C. guianensis* varies across studies, primarily due to variations in the proportions of fatty acids. Factors like the collection site and period, extraction technique, and raw material storage can all contribute to this variability. Despite this vulnerability, oleic and palmitic acids consistently emerge as the major fatty acids in andiroba oil, as summarized in Table 1.

Analytical approaches further illustrate this compositional variability. Although Alves et al. [29] and Araujo-Lima et al. [30] used the same analytical approach, Araujo-Lima et al. [30] identified minor fatty acids—including myristic (C14:0), margaric (C17:0), arachidic (C20:0), gondoic (C20:1), behenic (C22:0), and lignoceric (C24:0)—at values below 0.4%. Their results indicated that monounsaturated fatty acids account for about 50%, with saturated and polyunsaturated fractions at roughly 38% and 11%, respectively, across extraction methods such as dried seed without autoclaving, with autoclaving, and Soxhlet extraction. The study confirmed that these extraction processes did not notably alter the oil’s composition. Comparable findings have been reported by Gomes et al. [17], who identified 12 saponifiable compounds, and by Iha et al. [31], who reported a comparable fatty acid profile for *C. guianensis* using n-hexane extraction and AOAC 963.15 methodology. Leal et al. [32] similarly identified oleic acid as the dominant component. Consistent profiles were also described by Melo et al. [33] and Milhomem-Paixão [34]. Raspe et al. [35] further demonstrated subcritical n-propane and Soxhlet n-hexane extraction, concluding that temperature changes (25–45 °C at 40–80 bar) did not significantly impact lipid composition. Notably, n-propane extraction yielded higher concentrations of phenolic compounds while maintaining similar antioxidant capacity to conventional methods.

In addition to fatty acids, *C. guianensis* contains a 2–5% unsaponifiable fraction comprising triterpenes, steroids, coumarins, flavonoids, diglycerides, and notably limonoids (bioactive molecules also characteristic of the *Meliaceae* family) [32,36,37,38]. Limonoids are highly oxygenated modified terpenoids classified as tetranortriterpenoids. These compounds occur in both neutral (aglycone) and acidic (carboxylated, glycosylated) forms and are structurally derived from a 4,4,8-trimethyl-17-furanylsteroid precursor. Citrus limonoids characteristically contain a furan ring at C-17 (D-ring) and oxygenated functional groups at C-3, C-4, C-7, C-16, and C-17. Limonoids derived from the *Meliaceae* family demonstrate significant potential for environmentally sustainable pest control and exhibit a broad spectrum of biological activities [37]. Ambrozin et al. [38] identified seven limonoids of andiroba oil (17β-hydroxyazadiradione, gedunin, 6α-acetoxygedunin, 7-deacetoxy-7-oxogedunin, 1,2-dihydro-3β-hydroxy-7-deacetoxy-7-oxogedunin, methyl angolensate, and xyloccensin K) using multiple chromatographic techniques. Other researchers have identified a wide spectrum of limonoids in andiroba oil, as summarized by several studies [39,40,41,42,43,44,45,46,47,48,49,50,51,52].

Phenolic compounds further contribute to the antioxidant activity and biological properties of andiroba oil. Novello et al. [53] reported yields up to 35.67 mg/g of catechol under optimized extraction conditions (25 °C and 13 bar in n-butane solvent). Phenolic yields reported by Araujo-Lima et al. [30] in three samples were 10.34 ± 0.04, 9.50 ± 0.02, and 9.00 ± 0.03 mg/g catechol, while Leal et al. [32] found 13.4 ± 0.03 mg/g. Raspe et al. [35] demonstrated that n-propane extraction produced phenolic contents ranging from 26.5 to 41.4 mg GAE/100 g oil, up to 53% higher than Soxhlet-extracted oils. For a comprehensive overview of andiroba oil’s chemical composition, extraction variables, and principal bioactive constituents, detailed analyses are available in Dias et al. [23].

Collectively, these findings highlight substantial variability in the chemical composition of andiroba oil. The diversity and relative concentration of chemical constituents in plant oil samples can fluctuate markedly depending on genetic background, environmental conditions, and the particulars of collection and processing [54]. Additionally, the oil extraction method represents a significant source of variability, as parameters such as temperature and the use of specific solvents may promote the degradation of fatty acids [55]. Moreover, an important aspect to be considered in compound identification pertains to the solvents employed in chromatographic analyses. The choice of solvent may adversely affect the solubility and extractability of the target compound, thereby reducing analyte affinity and ultimately resulting in incomplete or insufficient extraction [56]. This underscores the need for standardized methodologies in its characterization and application.

**Figure 2:**
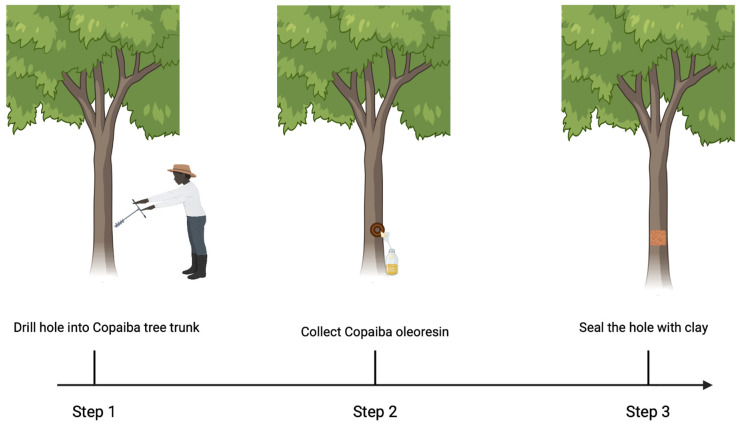
Traditional method for Copaiba oleoresin extraction. Step 1: A hole is drilled into the trunk of the Copaiba tree using a manual auger. Step 2: The oleoresin (a natural mixture of essential oils and resins) flows from the tree and is collected in a container. Step 3: After collection, the hole is sealed with clay to protect the tree and allow regeneration. (Created with biorender.com.)

*Copaiba Oil.* The extraction of copaiba oil from various species of *Copaifera* trees native to the Amazon rainforest reflects a practice that merges ancestral knowledge with modern sustainable techniques. Typically, a small incision is drilled into the trunk of a mature tree to access the internal canals where the oleoresin accumulates. This viscous mixture of essential oils and resins then flows spontaneously due to the internal trunk pressure and is collected in containers positioned at the base of the tree. Once the desired volume is obtained, the hole is carefully sealed, usually with clay, to prevent infections and allow natural healing (Figure 2). Individual trees can produce between 1 and 6 L of oleoresin per year, depending on age and environmental conditions. Notably, some *Copaifera* trees can live for over four centuries, highlighting the ecological importance of non-destructive extractions methods [57,58].

Copaiba oil is primarily composed of sesquiterpenes and diterpenes, corresponding to its volatile and resinous fractions, respectively. The chemical composition of these terpenoids varies considerably among species within the *Copaifera* genus, because of genetic diversity, geographic distribution, and environmental influences [9,25]. Among the sesquiterpenes, β-caryophyllene consistently appears as the major constituent and is widely recognized as a chemical marker due to both its prevalence and biological significance. Other relevant sesquiterpenes, including α-humulene, α-copaene, trans-α-bergamotene and β-bisabolene, also contribute significantly to the pharmacological properties of the oil. These components are detailed further in Table 2 [59]. In parallel, diterpenes comprise bioactive molecules such as copalic acid, kaurenoic acid and hardwickiic acid, which exhibit documented cytotoxic, anti-inflammatory and antimicrobial effects. Recent advances in analytical methodologies and nanotechnology have enabled not only the isolation but also the structural optimization of these compounds, paving the way for their incorporation into nanostructured delivery systems aimed at enhancing solubility, bioavailability and tissue-specific targeting [25]. This biochemical diversity reinforces the potential of copaiba oil as a multifunctional agent in pharmaceutical, veterinary and cosmeceutical applications.

In addition to its terpene-rich profile, copaiba oil contains a measurable fraction of fatty acid derivatives, which account for approximately 10.5% of its overall composition. The principal fatty acids identified include oleic acid (3.9%), linoleic acid (3.4%), palmitic acid (2.0%), and stearic acid (1.2%). Together, these components represent a balanced distribution across saturated (3.2% from palmitic and stearic acids), monounsaturated (3.9% of oleic acid), and polyunsaturated (3.4% from linoleic acid) fatty acid categories, contributing additional nutritional and functional properties to the oil [59].

### 3.2. Physicochemical Properties of Andiroba and Copaiba Oils

*Andiroba Oil.* Andiroba oil is characterized by a bitter taste [32], translucent light-yellow color, and a tendency to solidify below 25 °C [53,70,71]. Although environmental factors such as geographic origin and harvest season exert a relatively minor influence on its physicochemical properties compared to chemical composition, variables including extraction methods and raw material storage significantly affect key parameters such as pH and acidity [32,72]. Critical quality indicators, including parameters such as pH, acidity, and peroxide index, are crucial not only for assessing oil freshness and stability, but also for mapping oxidative changes, hydrolysis, and potential fermentation, all of which impact product quality [30,31,71]. The refractive index, which correlates with the degree of bond saturation, serves as an important parameter for comparing average molar mass and differentiating andiroba oil from others [73]. It typically ranges between 1.459 and 1.466 [30,72,74]. Density values for andiroba oil are generally reported around 0.905–0.92 g/cm^3^ [72,75], while viscosity typically falls in the range of 38.4 to 46.6 mm^2^/s [31,75].

Acidity values vary according to extraction and analysis methods, ranging from 2.3 to 36.1 mg KOH/g [24,26,31,72]. Similarly, the peroxide index, a proxy for oxidative degradation, ranges between 0.97 and 8.56 mEq/kg, tracking storage and extraction quality [24,30,32]. The saponification index, reflecting fatty acid chain length, spans 190–210 mg KOH/g [75].

Advanced analytical techniques, including nuclear magnetic resonance (NMR), differential scanning calorimetry (DSC), and thermogravimetric analysis (TGA), have provided further insight into the thermal stability of andiroba oil. These analyses also characterize its predominant triacylglycerols, such as Palmitic–Oleic–Oleic and Palmitic–Palmitic–Oleic, as well as minority compounds and the unsaponifiable fraction [70,76].

*Copaiba Oil.* Copaiba oil exhibits distinct physicochemical characteristics that vary according to its botanical origin and processing conditions. The oleoresin is insoluble in water and only partially soluble in alcohol. Visually, its appearance can range from transparent to opaque, with coloration varying from pale yellow to light golden brown, depending on the species and origin of the resin [77,78]. It is also characterized by a strong, sharp aroma reminiscent of coumarin, which reflects the complexity of its volatile profile constituents [79].

Reported pH values generally are between 5.27 and 5.53 [80], while density typically ranges from 0.9497 to 0.9690 g/cm^3^. Viscosity exhibits substantial variability, with reports ranging from 28.50 to 1707.73 mm^2^/s [81,82]. This broad dispersion cannot be attributed exclusively to extraction procedures, but rather reflects intrinsic differences related to the botanical source, including interspecific variation within the *Copaifera* genus, as well as differences in the relative proportions of volatile sesquiterpenes and resinous diterpene [78,82]. Additional factors, such as collection site, soil characteristics, oxidative state and storage conditions, contribute to observed heterogeneity among samples [71,77]. The refractive index, used as a marker for oil authenticity and purity, has been reported between 1.507 and 1.511 [77].

Acidity, an important parameter for evaluating freshness and quality, ranges from 50.6 to 55.87 mg KOH/g, while the saponification index varies from 62.63 to 98.40 mg KOH/g. These fluctuations suggest differences in the oil’s fatty acid compositions and the average chain length of its triglycerides [71,78]. Exposure to air promotes darkening of the resin and increases in both viscosity and density, factors that are critical for storage stability and shelf-life considerations [80]. Gas chromatography–mass spectrometry (GC-MS) analyses have consistently identified β-caryophyllene as the major sesquiterpene in copaiba oil, a compound linked to both its distinctive scent and a wide range of biological activities [9,83]. More recently, efforts to incorporate copaiba oil into nanoemulsion systems have demonstrated improved stability and bioavailability [83].

*Quality control.* Considering the variability reported for both copaiba and andiroba oils, the establishment of standardized quality-control parameters is essential to improve reproducibility and comparison across studies [30,32,35,54,59,84]. These parameters may include the use of characteristic chemical markers, such as β-caryophyllene for copaiba oil and limonoids for andiroba oil, together with defined physicochemical criteria, including acidity index, peroxide value, density, viscosity, refractive index and residual solvents [30,31,35,59]. Chromatographic fingerprinting, particularly based on gas chromatography profiles, may be used to ensure compositional consistency and detect potential adulteration [54,59]. In addition, stability-related criteria should include the monitoring of oxidative degradation and changes in key constituents during storage [35,59]. The adoption of such parameters would support more reliable characterization and contribute to the development of reproducible and regulatory-compliant formulations.

### 3.3. Pharmacological Activities of Andiroba and Copaiba Oils

*Andiroba Oil.* Andiroba oil has long been utilized in traditional Amazonian medicine, with ethnopharmacological records documenting its use in the treatment of coughs, convulsions, skin diseases, arthritis, rheumatism, ear infections, wounds, diarrhea, diabetes, bruises, and as an insect repellent [29,85]. Pharmacologically, the efficacy of andiroba oil is linked to its unique composition rich in limonoids, tetranortriterpenoids, fatty acid amides, and other bioactive compounds.

Notably, andiroba oil demonstrated potent anti-leishmanial effects, promoting parasite death at low doses without cytotoxicity to host cells. The authors suggested that the observed leishmanicidal activity may be attributed to the presence of sesquiterpenes and diterpenes, potentially acting through synergistic interactions between these compounds [86]. This activity was further evaluated by other authors using limonoid-rich fractions to determine their antileishmanial activity against promastigote and intracellular amastigote forms of *Leishmania amazonensis* [87].

Moreover, consistent with its extensive traditional use, the activity of andiroba oil against malaria-causing protozoa (*Plasmodium falciparum*) has also been investigated. Andiroba oil and its limonoid-rich fraction were subjected to antiplasmodial assays and demonstrated significant inhibitory activity in both in vitro [88] and in vivo [49] studies, exhibiting effects comparable to those of classical antimalarial agents, such as quinine and chloroquine. Additionally, the oil exhibits acaricidal activity against *Amblyomma nitens* and *Rhipicephalus sanguineus* adults, as well as efficacy in controlling *Boophilus microplus* infestations under in vitro conditions [14,89,90].

It also exhibits anticonvulsant activity; fatty acid amides derived from *Carapa guianensis* function as modulators of pentylenetetrazole-induced seizures via interaction with the GABA-A receptor [91]. Of particular interest is the antineoplastic activity of fatty acid amides derived from *Carapa guianensis*, which reduce viability, proliferation, and migration in C6 glioma cells dose-dependently while sparing normal glial cells, highlighting their therapeutic potential with minimal toxicity [90]. Additionally, andiroba oil has demonstrated the ability to decrease the viability of ACP02 gastric adenocarcinoma cells through apoptosis induction without exerting mutagenic effects [92].

Further investigations confirm the broad anti-inflammatory potential of andiroba oil [10,11,12]. Experimental investigations conducted in rats [10] and hamsters [11] with periodontitis and oral mucositis, respectively, reported a significant reduction in inflammatory cell infiltration following treatment with andiroba oil. Beyond preclinical models, its therapeutic potential was also evaluated in a clinical study involving children with leukemia affected by oral mucositis, in which andiroba oil was compared with low-level laser therapy. The findings revealed significantly superior therapeutic outcomes in the andiroba-treated group. These anti-inflammatory effects have been consistently attributed to the presence of bioactive compounds, particularly limonoids and triterpenes, in the oil composition [12]. Such attributes have also been observed in studies evaluating the immunomodulatory effects of limonoids extracted from the oil [39,40,45,52,93]. However, the anti-inflammatory potential of the major fatty acid components present in andiroba oil has been widely investigated. It is known that α-linolenic acid (ALA), one of the major compounds found in andiroba oil, is metabolized into eicosapentaenoic acid (EPA) and docosahexaenoic acid (DHA) through desaturation and elongation reactions of its fatty acid chain [94]. Furthermore, these fatty acids have been extensively investigated due to their inflammation-modulating properties [95].

**Figure 3:**
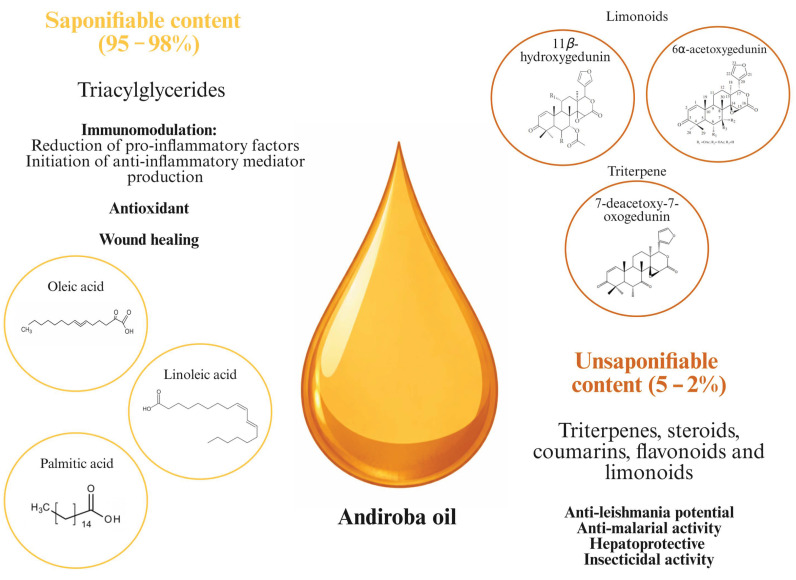
Schematic representation of the major triacylglycerides, limonoids and triterpenes found in andiroba oil, representing the most abundant or pharmacologically relevant constituents within its saponifiable (95–98%) unsaponifiable (5–2%) contents. Numerous other minor compounds are present in the oil but are not shown here. (Created with biorender.com.)

The pharmacological activities of andiroba oil are illustrated in Figure 3.

**Figure 4:**
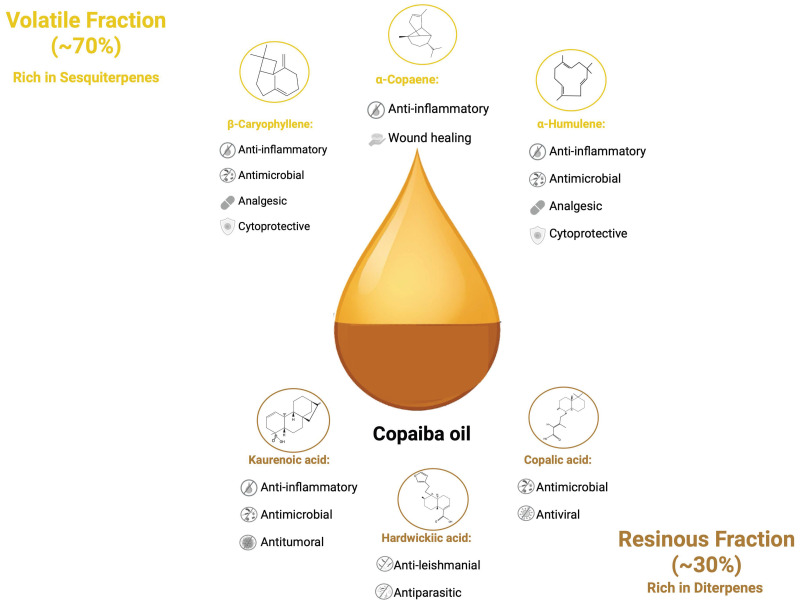
Schematic representation of the major sesquiterpenes and diterpenes found in copaiba oil, representing the most abundant or pharmacologically relevant constituents within its volatile (~70%) and resinous (~30%) fractions. Numerous other minor compounds are present in the oil but are not shown here. (Created with biorender.com.)

*Copaiba Oil.* Copaiba oil has long occupied a prominent position in traditional medicine, particularly among indigenous people of South America, owing to its use in treating a wide array of ailments [96]. Its therapeutic properties are closely tied to its complex chemical composition, which comprises a volatile fraction rich in sesquiterpenes and a resinous fraction dominated by diterpenes [97,98]. Although minor constituents such as phenolic compounds and monoterpenes are also present, the proportions and type of bioactive molecules can vary across different *Copaifera* species, influenced by both genetic factors and environmental conditions [97]. An overview of copaiba oil’s major sesquiterpene and diterpene constituents is present in Figure 4, highlighting their respective chemical classes and therapeutic roles.

Within the sesquiterpene fraction, β-caryophyllene (BCP) and α-humulene are particularly prominent due to their high abundance and robust pharmacological activity. BCP, in particular, has been shown to exert a wide range of effects, including antimicrobial, insecticidal, local anesthetic, antitumor, anti-inflammatory and anti-leishmal actions. In parallel, α-Humulene has been extensively studied for its potent anti-inflammatory effects and its inclusion in topical pharmaceuticals such as Acheflan^®^ [99,100]. While numerous studies have focused on the biological actions of these compounds in insolation, emerging evidence suggests that the full therapeutic potential of crude copaiba oil may derive from synergistic interactions between its multiple constituents [97].

The diterpene fraction contributes to its pharmacological profile and includes compounds such as copalic acid, kaurenoic acid, polyalthic acid hardwickiic acid. These molecules are associated with various therapeutic effects, including anti-inflammatory [69,100], antinociceptive [101] and antiparasitic [102,103]. Additionally, diterpenes from copaiba oil have demonstrated antimicrobial [104,105,106], antiviral [9], and antitumoral properties [107], expanding the potential applications of copaiba oil in both pharmaceutical and veterinary contexts.

More recently, copaiba oil has also been associated with immunomodulatory properties. As part of the broader class of Amazonian oils, it has been shown to regulate immune responses and suppress inflammation, effects largely attributed to its rich terpene content [21]. This complements findings from in vivo models, where copaiba oil was shown to promote re-epithelization and reduce inflammatory infiltration in lung tissue affected by allergic asthma [7]. These immunopharmacological attributes consolidate the oil’s status as a multifunctional agent with considerable relevance in drug discovery and natural product research.

Emerging studies continue to expand the therapeutic landscape of copaiba oil. For instance, Mello et al. [9] reported the bactericidal effects of copaiba oil in *Oreochromis niloticus* (Nile tilapia), along with health benefits following oral supplementation. Similarly, Silva et al. [108] demonstrated that copaiba essential oil possesses notable antibacterial and antibiotic enhancing effects against *Staphylococcus aureus*, particularly when combined with light-based technologies. Collectively, these findings underscore the broad-spectrum bioactivity of copaiba oil but also support its development into innovative drug delivery systems, such as nanoemulsion or lipid carriers, for enhanced clinical efficacy.

### 3.4. Applications of Andiroba and Copaiba Oils in Advanced Delivery Systems

Nanotechnology has emerged as a promising approach to overcome the limitations associated with the poor water solubility and bioavailability of lipophilic natural products such as andiroba and copaiba oils. Various advanced delivery systems, including lipid and polymeric nanoparticles (NPs), nanoemulsions (NEs), liposomes, and polymeric micelles have been developed to enhance the pharmacokinetic and pharmacodynamic profiles of these oils [109]. These nanocarriers offer numerous advantages over conventional oil formulations such as improved solubilization of hydrophobic compounds, sustained and controlled release of active ingredients; protection against chemical and enzymatic degradation of labile molecules; reduction in volatilization losses; and minimized side effects [100,101,102,103,104,105,106,107,108,109,110,111].

Among available nanoformulations, nanoemulsions are the most extensively studied due to their ability to disperse hydrophobic oils in aqueous media, enhance chemical stability, and ensure controlled drug release [112,113]. Recent work has further explored polymeric nanocapsules, showing favorable biosafety profiles in lung cell models, supportive of their use as phytotherapeutic medicine carriers [114]. Similarly, nanostructured lipid carriers (NLCs) utilize a hydrophilic surface and hydrophobic core to preferentially solubilize plant bioactive while improving stability under physiological conditions [112,113]. In addition to the most widely investigated nanoformulations, superparamagnetic iron oxide nanoparticles (SPIONs) formulated as biocompatible magnetic nanofluids containing andiroba and copaiba oils have been proposed as a new possibility for applications in medical imaging and cancer therapy [115]. Furthermore, aqueous suspensions of maghemite nanoparticles and colloidal emulsions containing magnetite nanoparticles previously coated with oleic acid have also been reported, using natural andiroba seed oil as the dispersing medium [116]. Nevertheless, only a limited number of studies have investigated these nanostructured systems using vegetable oils as dispersing or functional media.

*Andiroba Oil.* Despite its rich pharmacological properties, the fatty nature of andiroba oil presents formidable obstacles to its solubility in aqueous environments and challenges in oral absorption and intravenous delivery. However, several studies have investigated nanotechnology-based delivery of andiroba oil, with nanoemulsions emerging as predominant systems facilitating its therapeutic and cosmetic applications. Typically, these nanoemulsions are oil-in-water (O/W) systems containing 10–20% oil and stabilized with 0.5–2% surfactant. In these systems, bioactive compounds are solubilized, dispersed, or adsorbed within the internal nanostructure [109], and the main distinction between conventional emulsions and nanoemulsions lies in the energy input during preparation and the ability to reduce droplet size to the nanoscale.

Nanoemulsions containing andiroba oil have been shown to exhibit broad biological activity against pathogens affecting different species. Vaucher et al. [117] reported high activity of andiroba oil against *Paenibacillus* species—pathogens associated with bee infections—providing evidence that nanoemulsion-based systems enhance the oil’s bioactivity while preserving its volatile constituents. Similarly, Baldissera et al. [110] demonstrated that both conventional and nanostructured formulations of andiroba oil display pronounced in vitro trypanocidal activity against *Trypanosoma evansi*, with nanoemulsions exhibiting faster and more effective antiparasitic action compared to crude oil.

Beyond their activity against animal pathogens, andiroba oil nanoemulsions have also shown efficacy against vectors of high-impact human diseases, including dengue and leishmaniasis. Jesus et al. [118] demonstrated significant larvicidal activity against *Aedes aegypti* larvae, with bioactivity progressively increasing over time due to the gradual release of compounds from the nanoemulsions. Innovations include use of silk fibroin from *Bombyx mori* as an alternative emulsifier to create stable oil-in-water emulsions with high unsaturated fatty acid content, showing potent larvicidal effects against *Aedes aegypti* [74]. Furthermore, Moraes et al. [103] confirmed the leishmanicidal potential of andiroba oil nanoemulsions against *Leishmania infantum* and *Leishmania amazonensis*, reporting a significant reduction in infection levels in macrophage cultures treated with concentrations of 200 and 300 μg/mL.

Additionally, in in vitro studies involving nanotechnology, particularly in the context of cancer and immunomodulation research, andiroba oil-based systems are widely reported in the literature. Monteiro et al. [24] developed nanoemulsions that promoted accelerated keratinocyte migration and wound closure without inducing cytotoxicity. Borges et al. [119] formulated monodisperse NLCs combining semi-synthesized fatty acid amides from andiroba and silk fibroin, exhibiting selective cytotoxicity against murine breast cancer cells while sparing normal fibroblasts. Hybrid polymeric sponges incorporating poly(ε-caprolactone) and andiroba oil also showed promising wound healing and biocompatibility profiles [18]. Furthermore, regarding wound healing, according to the study by Fonseca et al. [16], andiroba oil was able to modulate the immune system; this was evidenced by its ability to dampen leukocyte recruitment, enhance phagocytic activity, and regulate key cytokines and interleukins involved in the inflammatory response. It also supported tissue repair by reducing apoptotic activity and promoting processes such as reepithelialization and angiogenesis, with advanced formulations, such as nanoemulsions, films, and gels, showing even greater immunomodulatory effects than the crude oil. Regarding studies in vivo, Melo et al. [33] evaluated andiroba oil nanoemulsions for the mitigation of doxorubicin (DOX) induced hepatic and renal lesions in mice, demonstrating enhanced protective effects and improved pharmacokinetic profiles.

*Copaiba Oil.* The pharmacological application of copaiba oil is limited by its low aqueous solubility and chemical instability, which have driven the development of nanotechnology-based delivery systems to improve stability, control release, and enhance therapeutic performance [112,113,114,120].

Initial investigations have highlighted the potential of copaiba-oil-based nanocarriers against pathogens affecting non-human species and disease vectors. Nanoemulsions containing copaiba oil have demonstrated insecticidal and antiparasitic activity, with enhanced efficacy attributed to improved dispersion and protection of volatile bioactive compounds [121]. These systems have shown increased activity when enriched with α-copaene, a sesquiterpene associated with antiparasitic effects.

Subsequently, several studies have explored the activity of copaiba oil nanoformulations against human pathogens in in vitro models. Copaiba nanoemulsions have exhibited significant antifungal activity against *Paracoccidioides* spp., offering advantages over crude oil, including improved stability and the possibility of alternative administration routes such as inhalation and intravenous delivery [106]. In the field of virology, nanoemulsions loaded with copaiba oil inhibited Zika virus replication by approximately 80% at non-cytotoxic concentrations [122].

The therapeutic potential of copaiba oil nanoformulations has also been validated in animal models. In murine models of cutaneous leishmaniasis, nanoemulsions incorporating copaiba oil resulted in a significant reduction in lesion size and parasite burden, demonstrating improved efficacy compared to non-nanoencapsulated formulations [103]. Additionally, the anti-inflammatory activity of copaiba oil nanoemulsions has been confirmed in vivo, with studies reporting reduced paw edema in animals treated with copaiba-loaded hydrogels, supporting their applicability in topical inflammatory conditions [102].

Beyond improving solubility and stability, nanocarriers may also influence how bioactive compounds from natural oils behave in biological systems. These systems can modify biodistribution patterns, for example, by increasing circulation time, improving tissue accumulation, and facilitating cellular uptake through endocytic pathways [123,124].

It is also important to consider that the composition of nanocarriers can influence the incorporation of compounds from complex oil matrices, such as those found in copaiba and andiroba oils. Factors such as the oil phase, surfactant type and carrier structure may favor the solubilization of specific constituents. As a result, the chemical profile of the formulation may differ from that original oil, which can contribute to variations in biological activity [109,114].

The key physicochemical, compositional, pharmacological, and delivery system attributes of copaiba and andiroba oils are summarized and compared in Table 3.

The major studies on delivery nanosystems of copaiba and andiroba oils are comparatively summarized in Table 4, emphasizing the nanocarrier type, experimental application, and the main therapeutic or technological findings reported for each system.

Substantially, the studies summarized in Table 4 indicate that nanoemulsions are the most versatile and established strategy for encapsulating both andiroba and copaiba oils, mainly because they combine relatively simple production, efficient dispersion of lipophilic constituents, and recurrent gains in biological response, including antiparasitic, antiviral, antifungal, larvicidal, and wound healing effects [21,74,121,122]. However, their performance depends primarily on surfactant composition, droplet size control, and storage conditions, which may limit long-term translational robustness despite the favorable stability reported in some formulations [23,24].

In contrast, polymeric nanocapsules and nanostructured lipid carriers (NLCs) appear to offer more sophisticated control over encapsulation and interfacial interactions, with the added advantage of improved biosafety, selective cytotoxicity, and greater structural protection of volatile or oxidation prone compounds, making them especially attractive for pulmonary and oncologic applications [25]. Nevertheless, these systems are still supported by fewer studies and often involve greater formulation complexity, which may hinder scalability and reproducibility [114,119].

Hybrid systems such as fibroin-stabilized polymeric films broaden the functional scope by reducing dependence on synthetic surfactants and enabling local, sustained delivery in tissue repair, but their therapeutic performance is more formulation-specific and less broadly validated across models [18,26,74]. In general, the comparative evidence suggests that nanoencapsulation consistently enhances the pharmacological utility of both oils, yet the current literature also reveals a balance between technological simplicity and functional sophistication. Nanoemulsions are the most consolidated platform for boosting bioactivity and improving colloidal stability, whereas NLCs, nanocapsules, and hybrid matrices may provide superior protection and targeting, although they still require stronger standardization and more robust comparative studies before their advantages can be generalized [24,25,123].

In addition, an important limitation across the currently available studies is that most investigations remain restricted to in vitro evaluations, with relatively few reports advancing to more robust and biologically complex in vivo models, which are essential to confirm therapeutic efficacy, biodistribution, safety, and translational applicability of these nanostructured systems.

## 4. Conclusions

This review provides a comprehensive analysis of the chemical compositions, physicochemical properties, and pharmacological activities of copaiba (*Copaifera* spp.) and andiroba (*Carapa guianensis*) oils, with particular emphasis on the role of nanotechnology-based delivery systems in overcoming the inherent limitations of these lipophilic substances. Although both oils are deeply rooted in traditional Amazonian medicine and exhibit broad therapeutic potential, their integration into modern biomedical applications remains relatively limited outside local and ethnopharmacological contexts.

Copaiba and andiroba oils share a broadly similar fatty acid profile, predominantly composed of oleic, linoleic, palmitic, and stearic acids. However, they differ significantly in their non-saponifiable fractions. Specifically, copaiba oil is enriched in sesquiterpenes and diterpenes, which underpin many of its pharmacological effects, whereas andiroba oil contains abundant triterpenes, notably limonoids, underpinning its bioactivities. Despite these compositional differences, both oils exhibit comparable physicochemical properties and overlapping biological effects, including anti-inflammatory, insecticidal, antimicrobial, and potential antileishmanial properties.

Over the past decade, nanotechnology-based delivery systems, especially nanoemulsions and lipid-based systems, have emerged as a promising strategy to enhance the stability, bioavailability and therapeutic efficacy of copaiba and andiroba oils. Studies published since 2013 demonstrate that nanoformulations not only improve the delivery of bioactive constituents but also broadens the scope of applications, positioning these oils as both active agents and functional platforms for advanced drug delivery systems. Furthermore, other nanostructured systems, such as liposomes, hexosomes and nanofibers, may also be explored, as well as any other nanoformulation capable of incorporating oily substances.

In addition to improving physicochemical properties, nanocarriers may influence biodistribution, cellular uptake and the mechanism of action of bioactive compounds. Furthermore, the composition of these systems can affect the selective incorporation of constituents from complex natural oils, which may impact their pharmacological effects.

Nevertheless, several limitations must be acknowledged. As an integrative review, this work is constrained by the heterogeneity of available studies, including variations in oil composition, extraction methods, formulation strategies and experimental models. In addition, the majority of evidence supporting the pharmacological potential of nanoencapsulated copaiba and andiroba oils derives from in vitro assays and animal studies, with a marked lack of well-designed clinical trials. This limits direct translation to human applications and underscores the need for more standardized and comparable research approaches.

Despite growing body evidence, several research gaps remain for both copaiba and andiroba oils. There is still considerable variability in reported chemical composition and physicochemical properties, often associated with differences in species identification, geographic origin, extraction methods, and storage conditions. This variability limits comparability across studies and highlights the need for standardized analytical and processing protocols. In addition, most available data are based on in vitro or short-term in vivo studies, with limited information regarding long-term toxicity, pharmacokinetics and chronic exposure. Another important aspect that remains poorly understood is the contribution of individual compounds versus potential synergistic effects within these complex mixtures.

Safety and toxicity considerations also require further attention. While nanoencapsulation is frequently proposed as a strategy to reduce irritation, volatility or instability associated with crude oils, the long-term safety of nanosystems, including the potential effects of surfactants and other excipients, remains insufficiently explored. Comprehensive toxicological evaluations and extended safety studies are therefore essential to ensure that advantages of nanoformulation outweigh potential risks.

In conclusion, this review consolidates current knowledge on copaiba and andiroba oils and highlights the role of nanotechnology in enhancing their therapeutic applications. At the same time, it addresses critical gaps related to variability, safety and clinical translatability. Future research should prioritize standardized characterization, robust toxicological evaluation and well-designed clinical studies, alongside sustainable sourcing practices, to fully harness the medicinal potential of these Amazonian oils within a modern biomedical framework. Nevertheless, Amazonian oils have thus far demonstrated promising preclinical outcomes, although important translational barriers still remain.

## 5. Limitations and Future Directions

Despite the significant advances in the development of nanoformulations containing Amazonian oils, several limitations remain, particularly regarding andiroba and copaiba oil-based nanosystems, defining key opportunities for future research.

### 5.1. Toxicity and Long-Term Stability

Most studies involving nanoformulations of andiroba and copaiba oils focused on short- to medium- term physicochemical stability, often limited to a few months. Comprehensive accelerated and long-term stability assessments, especially under conditions that simulate real-world storage and use, are still limited. Furthermore, although both oils are widely regarded as biocompatible in their crude forms, the chronic toxicity and long-term safety of nanoformulations remain insufficiently characterized. This concern is particularly relevant given the inclusion of surfactants and other excipients, which may influence biocompatibility and toxicity profiles over prolonged exposure periods [123].

### 5.2. Clinical Translation and Human Trials

The therapeutic potential of nanoformulations containing andiroba and copaiba oils has been predominantly demonstrated in vitro systems and animal models, showing promising anti-inflammatory, antiparasitic, antiviral and antifungal activities. However, well-designed, randomized clinical trials in humans remain scarce for both oils. Transitioning from preclinical models to clinical evaluation is essential to confirm efficacy, establish safety margins and define appropriate dosing regimens, representing a critical step toward clinical and pharmaceutical translation [124].

### 5.3. Standardization and Regulatory Challenges

A major limitation lies in the lack of standardization of nanosystems derived from andiroba and copaiba oils. Variability in oil composition due to botanical origin, geographic location and extraction methods, combined with differences in formulation strategies and analytical characterizations protocols, hampers direct comparison among studies. The establishment of standardized quality control parameters and validated analytical methodologies is therefore imperative to meet regulatory requirements imposed by agencies such as ANVISA, FDA and EMA. Strengthening methodological rigor and harmonization in study design and reporting will be fundamental for advancing the regulatory approval, clinical translation and industrial scalability of nanosystems derived from both oils [125].

## Figures and Tables

**Table 1:** Fatty acid compositions of *Carapa guianensis*.

Mainly Fatty Acids					Studies
Oleic Acid (C18:1)	Palmitic Acid (C16:0)	Stearic Acid (C18:0)	Linoleic Acid (C18:2)	Analytical Techniques	
57.0%	24.3%	10.2%	5.9%	GC-MS; GC-FID	[29]
49.9% (a) 49.7% (b) 49.56% (c)	27.7% (a) 27.5% (b) 28.3% (c)	9.3% (a) 9.5% (b) 9.1% (c)	9.6% (a) 9.8% (b) 9.6% (c)	GC-MS; GC-FID	[30]
47.3%	31.5%	7.1%	9.0%	GC-MS	[17]
57.8%	25.3%	10.5%	5.9% (C18:2cis/cis9,12)	GC-MS	[31]
85.6% (316.9 mg/g)	0.1% (0.4 mg/g)	0.2% (0.6 mg/g)	0.2% (0.7 mg/g)	GC-FID	[32]
39.1% (ω9)	33.2%	4.7%	16.9% (ω6)	GC-MS	[33]
55.2%	37.9%	0.0%	4.2%	GC-MS	[34]
47.6%	28.6%	8.8%	9.6%	GC-MS; NMR	[24]
46.9% (c) 46.0% (d) 45.7% (e) 46.3% (f) 46.2% (g) 45.3% (h)	31.0% (c) 29.0% (d) 30.1% (e) 28.9% (f) 28.4% (9g) 28.2% (h)	12.1% (c) 11.3% (d) 11.1% (e) 11.2% (f) 11.6% (g) 12.2% (h)	5.5% (c) 9.2% (d) 8.8% (e) 9.2% (f) 9.3% (g) 9.6% (h)	GC-MS	[35]

Note: sample extracted by (a) dried seed without autoclaving; (b) with autoclaving; (c) Soxhlet; (d) 25 °C and 40 bar; (e) 25 °C and 80 bar; (f) 45 °C and 40 bar; (g) 45 °C and 80 bar; (h) 35 °C and 60 bar. Abbreviations: GC-FID: Gas chromatography with flame ionization detection; GC-MS: gas chromatography-mass spectrometry; NMR: nuclear magnetic resonance.

**Table 2:** Principal sesquiterpenes and diterpenes identified in copaiba oil across different *Copaifera* species of copaiba oil.

Genus	Major Sesquiterpenes		Major Diterpenes		Analytical Techniques	Studies
*C. cearensis*	β-caryophyllene 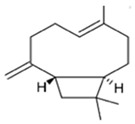	19.7%	Chrolechinic acid 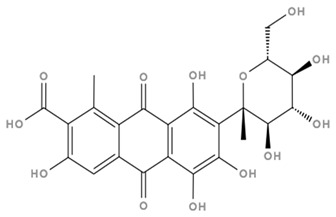	11.3%	CC; GC-FTIR; HPLC; IR; NMR; EI-MS	[60,61]
	Ledol 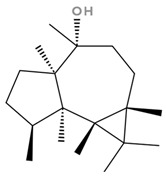	*				
	Caryophyllene oxide 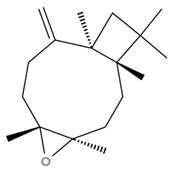	*				
*C. dukei*	Caryophyllene oxide 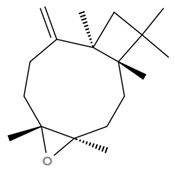	*	Kaurenoic acid 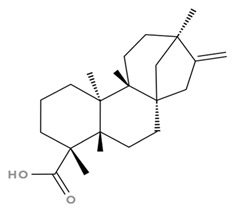	*	GC; GC-MS	[62,63,64]
	α-bergamotene 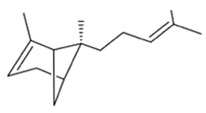	*	Copalic acid 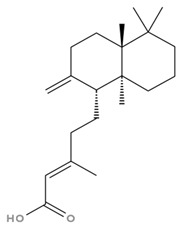	*		
	β-elemene 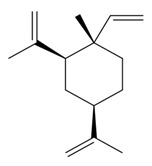	*	Polyalthic acid (labdane-type) 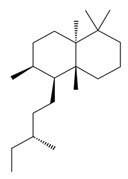	*		
	α-selinene 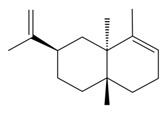	*	Hardwickiic acid 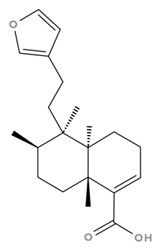	*		
	β-bisabolene 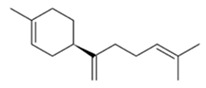	*				
*C. glycycarpa*	β-caryophyllene 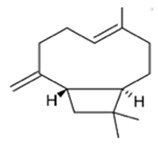	18.53%	Kaurenoic acid 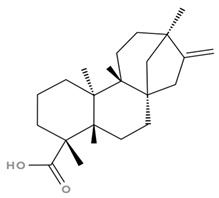	0.98%	HSCCC; NMR; MS	[65]
	β-bisabolene 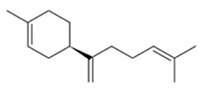	8.25%	Polyalthic acid (labdane-type) 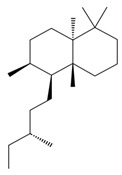	19.73%		
*C. guyanensis*	Caryophyllene oxide 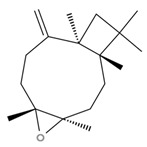	19.1%	Kaurenoic acid 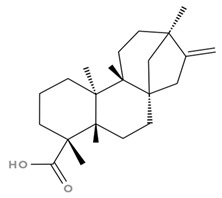	17.5%	GC; GC-MS	[62]
			Hardwickiic acid 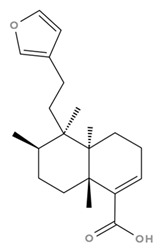	11%		
			Polyalthic acid (labdane-type) 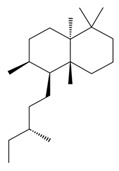	10.6%		
*C. langsdorffii*	β-caryophyllene 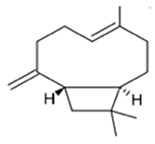	18.53%	Kaurenoic acid 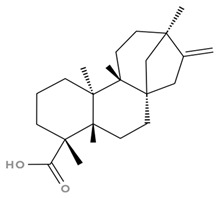	44.3%	GC; GC-MS	[54,66,67]
			Hardwickii acid 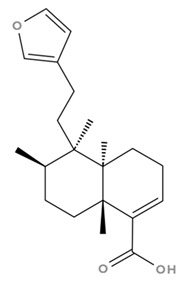	8.2%		
*C. lucens*	β-caryophyllene 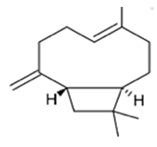	1.8%	Polyalthic acid (labdane-type) 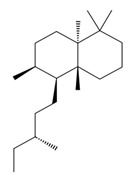	69.2%		
			Copalic acid 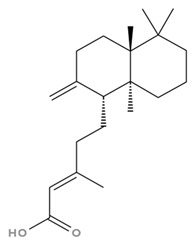	11.1%		
*C. martii*	α-copaene 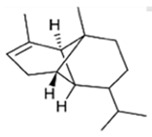	36.4–51.2%	*	*		
	δ-cadinene 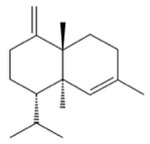	17.7–17.3%				
*C. paupera*	α-copaene 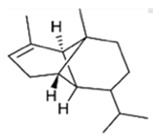	42.5%	Copalic acid 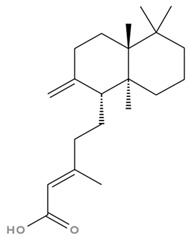	*	CC; GC; GC-MS; NMR; MS	[68,69]

Note: * Data not available or not reported in the cited study. Abbreviations: CC: column chromatography; GC: gas chromatography; GC-FTIR: gas chromatography–Fourier transform infrared spectroscopy; GC-MS: gas chromatography–mass spectrometry; HPLC: high-performance liquid chromatography; IR: infrared spectroscopy; NMR: nuclear magnetic resonance; EI-MS: electron ionization mass spectrometry; HSCCC: High-speed counter-current chromatography; MS: mass spectrometry.

**Table 3:** Comparative overview of the physicochemical properties, chemical compositions, traditional uses, pharmacological activities, advanced delivery systems, and clinical potentials of copaiba and andiroba oils. Data summarizes differences and similarities tied to their botanical origin, regional extraction methods, and bioactive profiles.

Attribute	Copaiba Oil	Andiroba Oil
Source Species	*Copaifera* spp.	*Carapa guianensis*
Region & Processing	Amazon, stem tapping, oleoresin extraction	Amazon, seed pressing, oil extraction
Physical Appearance	Pale yellow to golden brown, strong aroma	Light yellow, bitter taste, translucent, solidifies <25 °C
pH	5.27–5.53	5.84
Density	0.9497–0.9690 g/cm^3^	~0.9054 g/cm^3^
Acidity (mg KOH/g)	50.50–55.87	8.31–36.1
Major Fatty Acids	Oleic (3.9%), Linoleic (3.4%), Palmitic (2.0%), Stearic (1.2%)	Oleic (~47–57%), Palmitic (~24–31%), Stearic (~7–12%), Linoleic (~6–17%)
Volatile/Resinous Compounds	Sesquiterpenes (β-caryophyllene, humulene), Diterpenes (kaurenoic, copalic, hardwickiic acids)	Limonoids, triterpenes, saponifiable fatty substances, steroids, flavonoids, phenolics
Main Bioactive Compounds	β-caryophyllene, humulene, kaurenoic acid, copalic acid	Gedunin, methyl angolensate, linoleic, oleic, palmitic acids, catechol
Traditional Applications	Anti-inflammatory, antitumor, wound healing, antimicrobial, pain, insect repellent	Insect repellent, anti-inflammatory, analgesic, wound healing, skin, diabetes, arthritis, ear infections
Pharmacological Activities	Anti-inflammatory, antitumor, antiparasitic (leishmania), antimicrobial, antiviral, antifungal, antinociceptive	Anti-inflammatory, wound healing, antileishmanial, anticonvulsant, antineoplastic, anti-arthritic, insect repellent, antimicrobial
Advanced Delivery Systems	Nanoemulsions, nanostructured lipid carriers, micelles, hydrogels, polymeric nanocapsules	Nanoemulsions, nanocapsules, lipid carriers, PCL hybrid films, silk fibroin systems
Clinical/Therapeutic Potential	Enhanced bioavailability, topical and systemic use, potential for controlled-release drug delivery	Improved healing and efficacy, reduced cytotoxicity, tailored nanotech solutions for oral, topical, systemic therapies

**Table 4:** Advanced delivery systems for copaiba and andiroba oil that are currently under development.

Delivery Systems	Oil	[oil] % (p/v)	Surfactant	Size (nm)	PDI	ZP (mV)	Stability	Biological Assay	Key Findings	References
NE	AOR	0.2	Soy lecithin	205.7	0.29	−4.16	120 days at 4 °C	In vitro Scratch assay in keratinocytes; cytotoxicity	Increased effectiveness (wound closure) compared to non-encapsulated oil; biocompatible system; promising for wound dressings.	[24]
	AEO CEO	10.0	Span^®^80; Tween^®^20	192.4; 211.5	0.12; 0.14	−56.4; 47.1	Unspecified	In vitro Antimicrobial activity and toxicity	Encapsulation promotes preservation of volatile constituents; increased bioactivity of the oil; acceptable toxicity.	[118]
	AEO	10.0	Span^®^80	240.0	−55.4	0.15	Unspecified	In vitro Trypanocidal activity	Improved trypanocidal action compared to non-encapsulated oil	[110]
	COR	20.0	Span^®^80 Tween^®^20; MTC	101.0 (HPH) 251.0 *	0.08	−31.8 (HPH) −36.1 *	30 days at 4 °C	Unvalued	Preparation method influences physicochemical characteristics; MCT strategy for fixing the volatile portion of the oil.	[113]
	AOR	0.5	Polysorbate 80	150.0	0.13	−24.2	Unspecified	In vitro larvicidal activity	High larvicidal activity	[118]
	AO	9:1	Polysorbate 80:Span^®^80	Unspecified	Unspecified	Unspecified	Unspecified	In vivo Evaluation of lesions caused by doxorubicin	Reduction in liver and kidney damage.	[33]
	AOR COR	10.0 10.0	Tween^®^80	88.2 76.1	0.16 0.14	−3.9 −2.5	90 days at 25 °C	In vitro Activity against promastigotes of *L. infantum* and *L. amazonensis* In vivo Evaluation of leishmanicidal activity in models of visceral and cutaneous leishmaniasis	Reduction in infection levels in macrophages; Reduction in parasite load in the liver and spleen.	[103]
	COR	5.0	Polysorbate 80	145.2	0.37	Unspecified	Unspecified	In vitro larvicidal assay against *Aedes aegypti*	showed promising residual larvicidal effect, suggesting utility for vector control	[114]
	COR	0.2	Pluronic F-127	34.9	0.12	Unspecified	Unspecified	In vitro Antifungal activity against *Paracoccidioides* spp.	Exhibits significant antifungal activity; Additive effect in combination with amphotericin B; Alternative routes, such as inhalation or parenteral administration.	[106]
	COR	~0.2	Unspecified	152.3	0.23	−29.5	30 days at 4 °C	In vitro Cytotoxicity of VERO E6 cells; Antiviral activity against *Zika* virus.	Increased intracellular uptake; at 180 µg/mL, the system maintained acceptable cell viability; inhibited viral replication by approximately 80%; exhibiting antiviral potential.	[122]
	Balsam copaiba oil	0.1–0.5	Tween^®^80	215.8–398.5	0.1–0.2	−6 to −8	1 to 2 years 4 °C	In vitro preliminary toxicity in *C. elegans*	Using low levels of surfactant, NE remained stable; tolerated pH variations and thermal stress; and exhibited low toxicity, except at the highest concentration tested.	[83]
	COR	20.0	Span^®^80	253.9	0.05	−31.3	Unspecified	In vitro Permeation/retention study	Enhanced skin permeation and retention of β-caryophyllene compared to non-encapsulated oil.	[120]
	COR	10.0	Tween^®^20 and Span^®^80	≥114.9	≥0.29	−24.4	Unspecified	In vitro Evaluation of antileishmanial activity	Increased antileishmanial activity against promastigotes of *L. amazonensis* and *L. infantum*.	[121]
NLC	AEO	5.0	Lipoid^®^ S-100; Tween^®^80	183.0	0.16	−22.9	90 days at 4 °C and 25 °C	In vitro Cytotoxicity in breast cancer MCF-7	Optimization and functionalization of the NLC surface improved the interaction with albumin; Increased cytotoxicity against MCF-7 cells.	[25]
	Fatty acid amides	1.0	Polysorbate 80	131.0	0.2	−8.3	30 days, temperature not specified.	In vitro Antitumor activity against 4T1 cells	Selective cytotoxicity for breast cancer cells	[119]
PCL film	AO	1.7–2.7	not applicable	not applicable	not applicable	not applicable	thermal stability	In vitro Toxicity against L929; Absorption capacity of biological fluids.	Biocompatible; promising for wound care.	[18]
NC	CO	1.6	Tween^®^80; Span^®^60	215.0	0.15	−18.0	30 days at 25 °C	In vitro Biocompatibility against erythrocytes; Cytotoxicity and genotoxicity tests against lung cells	Biocompatible; potential application via respiratory route.	[114]

Note: * spontaneous emulsification. Abbreviations: NE (nanoemulsion); NLC (nanostructured lipid carrier); NC (nanocapsule); AOR (andiroba oleoresin); AO (andiroba oil); CO (copaíba oil) AEO (andiroba essential oil); CEO (copaiba essential oil); COR (copaiba oleoresin); PDI (polydispersity index); ZP (zeta potential); HPH (High-Pressure Homogenization).

## Data Availability

No new data were generated or analyzed in this study. All information is derived from previously published studies, which are cited within the manuscript.
